# Screen exposure in early childhood and maternal behaviors: a cross-sectional study from Turkey

**DOI:** 10.1007/s11845-026-04322-1

**Published:** 2026-04-07

**Authors:** Zekiye Temizkan, Hatice Tuba Akbayram

**Affiliations:** Department of Family Medicine, Gaziantep University Faculty of Medicine, 27310 Gaziantep, Turkey

**Keywords:** Child, Computer, Mother, Smartphone, Tablet, Television

## Abstract

**Background:**

The first two years of life are critical for a child’s development, requiring optimal parental interaction and care, while limiting screen exposure is essential to prevent potential cognitive and socio-emotional delays.

**Aims:**

This study examined maternal use of digital devices during early childhood care and explored factors associated with children’s screen exposure.

**Methods:**

A cross-sectional study was conducted between February 1 and June 1, 2023, in three family health centers in Gaziantep. Face-to-face surveys were administered to 256 mothers with children aged 4 to 24 months.

**Results:**

A total of 60.5% of children were exposed to television daily (44.1% < 1 h, 16.4% ≥ 1 h), followed by 53.1% to smartphones (41.4% < 1 h, 11.7% ≥ 1 h) and 21.9% to computers/tablets (17.6% < 1 h, 4.3% ≥ 1 h). Mothers most frequently used smartphones to facilitate childcare. Screen exposure during feeding was reported in 40.7% for television (34.4% rarely/sometimes, 6.3% most of the time/always), 21.9% for computers/tablets (17.2% rarely/sometimes, 4.7% most of the time/always), and 46.1% for smartphones (37.1% rarely/sometimes, 9.0% most of the time/always. Children whose fathers demonstrated high paternal involvement in childcare exhibited significantly lower rates of television, smartphone, and computer/tablet use.

**Conclusions:**

The findings revealed that a significant proportion of mothers used digital technological devices during childcare, and that higher paternal involvement in childcare may have a potential mitigating effect on early screen exposure in children.

## Introduction

The first two years of life are a critical period for children, requiring optimal nutrition and care and characterized by rapid physical, cognitive and social development [1]. In this process, the most valuable gift a child can receive is parental love, time, attention and support [2]. Interaction with parents starts before birth, strengthens with gestures, smiles, crying, mutual glances and vocalizations, and continues throughout childhood. The quality of this early interaction is an important factor for the child’s healthy development and protection from diseases [3]. In this period, the attachment relationship with the mother, who is the primary caregiver of the child, is one of the cornerstones of human development with profound implications for the well-being of mother and child [4].

In recent years, in addition to more traditional forms of media such as television, digital media such as tablets and smartphones have been used as important tools in the daily lives of families [5]. Families use these technological devices as “digital pacifiers” or “digital caregivers” to calm, nurture and entertain their children, and these devices are frequently preferred in almost every activity of the child, such as dining table and bathroom [6]. However, there are some concerns that the use of screen technology in young children may harm their cognitive development [7]. Possible dangers of screen exposure in children include delayed cognitive and language development, attention deficit, impaired social interaction, aggression and violent behavior disorders, sleep problems and obesity [8]. Nevertheless, the literature emphasizes that not all forms of screen exposure are equally harmful, and that context, content, and parental involvement may also have positive influences on developmental outcomes. In particular, interactive viewing of age-appropriate educational content under parental supervision can support children’s language development, attention, and learning skills [9]. Moreover, interactive applications and e-books that provide responsive feedback have been shown to promote vocabulary acquisition, early literacy, and sustained attention in young children [10]. The World Health Organization (WHO) recommends no screen exposure for children under the age of two [11], and the American Academy of Pediatrics (AAP) recommends no screen exposure for children between 0–18 months except for video chatting [12].

In today’s digital age, exposure to and use of digital devices by young children under the age of two is increasing [6]. Children who used to watch television at four years of age are now using digital devices independently at four months of age [13]. A recent systematic review and meta-analysis study reported that three-quarters of children under two years of age do not meet screen time guidelines [14].

Although there are similar studies in the literatüre [15, 16], there is limited recent data in Turkey that comprehensively examines screen exposure and maternal behaviors of children in this age group. This study investigated maternal behaviors concerning the use of digital technological devices during early childhood feeding and care, and examined children’s screen exposure in this context. Furthermore, potential factors influencing children’s screen exposure, such as maternal technological device use and demographic characteristics, were explored.

## Methods

### Study design and participants

This cross-sectional study was conducted in Gaziantep, Turkey, between February 1, 2023, and June 1, 2023**.** Gaziantep is a prominent city in Turkey, known for its industrial infrastructure and socioeconomic development. To ensure socioeconomic diversity, participants were recruited from three family health centers within Gaziantep province**,** which were purposively selected based on the predominant income levels of their served populations (low, middle, and high).

Mothers who met the inclusion criteria and presented to the family health centers for any reason (e.g., routine child follow-up, vaccination, or for a health problem in themselves or their child) were provided with information about the study and invited to participate. The inclusion criteria were: being 18 years of age or older, having a child aged 4–24 months, being able to speak Turkish, and willingness to participate in the study. The exclusion criterion was having a diagnosed psychiatric disorder or currently using psychiatric medication.

A pilot study was conducted with 10 mothers to assess the feasibility, clarity, and acceptability of the questionnaire; modifications were made as necessary. These 10 mothers were not included in the final sample.

A total of 263 mothers who met the inclusion criteria were administered the questionnaire. None of the participants reported a history of psychiatric illness or current use of psychiatric medication. Seven mothers who did not respond to all questionnaire items were excluded from the study. The final sample consisted of 256 mothers. Since the number of mothers who declined to participate was not recorded, the response rate could not be calculated.

### Questionnaire form and data collection

The questionnaire, developed by the researchers based on a literature review, included items on maternal and child demographics (e.g., mother’s age, education, employment, income, number of child; child’s age, gender), perceived difficulty of childcare, availability of caregiving support, maternal digital device use during childcare, and the child’s screen time. Paternal involvement in childcare was assessed with the question, “Does your husband help with the feeding and care of your baby?”, with response options categorized as: a) never/rarely, b) sometimes, and c) most of the time/always. Paternal involvement in childcare was categorized as low (never/rarely/sometimes assists) and high (most of the time/always assists).

In this study, screen time was defined as the time spent using a television, smartphone, tablet or computer [17]. The duration of mothers’ use of these devices (regardless of whether they were with their child or not) was questioned. The responses were categorized as follows: a) None – Less than 1 h, b) More than 1 h but less than 2 h, c) 2 h or more. Mothers were asked to estimate their children’ average daily screen time for smartphones, televisions, and computers/tablets. The responses were categorized as: a) Does not watch, b) Less than 1 h, c) 1 h or more. Mothers were asked, "Which technological device do you use most to facilitate the feeding and care of your baby?". The responses were categorized as “a) television, b) smartphone c) tablet d) computer e) none. Mothers were asked whether they fed their children while exposing them to television, smartphones, or computers/tablets. The responses were categorized as follows: a) never b) rarely c) sometimes d) most of the time e) always.

The final question of the survey inquired whether the mothers had been informed by a physician about the harmful effects of screen exposure on their child’s health. The survey was administered to mothers using a face-to-face interview technique.

### Ethics

Before the study, physicians working at the designated family health centers were informed about the research, and their permission was obtained. Approval for the study was granted by the Gaziantep University Clinical Research Ethics Committee (Ethics Committee Approval No: 2022/329, Date:28.09.2022) and the Provincial Health Directorate. Mothers were informed about the study, and both verbal and written consent were obtained.

### Statistical analysis

SPSS version 25.0 package program was used in the evaluation of the data. Descriptive statistics were presented as frequency and percentage for categorical variables, and as mean ± standard deviation for the continuous variable. The normality of continuous variables was assessed using the Shapiro–Wilk test and histogram. The chi-square test was used to assess the associations between children’s screen use/exposure and factors such as maternal demographic characteristics, child age, maternal use of digital devices and paternal involvement. Variables showing statistically significant associations in bivariate analyses were further examined using logistic regression analysis. Statistical significance was set at *p* < 0.05.

## Results

The participants’ mean age was 30.6 ± 4.7 years. Among the participants, 37.9% were university graduates, 48.4% were employed, and 37.5% had a high income. The majority (60.5%) had only one child. The mean age of the children was 14.3 ± 7.1 months, and 50% were male. Approximately 23.4% of mothers reported significant difficulty in feeding and caring for their child, while 39.1% noted that fathers were actively involved in childcare most of the time/always. Furthermore, 19.1% of the participants had a dedicated caregiver for childcare (Table 1).

**Table 1:** Demographic characteristics of mothers and childs

Characteristics		*n*	%
Mean age of mothers (years): 30,6 ± 4,7			
Age category of mothers	< 30 years	111	43.4
	≥ 30 years	145	56.6
Mother’s education	Illiterate	1	0.4
	Primary/Secondary school	73	28.5
	High school	85	33.2
	University	97	37.9
Mother’s employment	Employed	124	48.4
	Homemaker	132	51.6
Family income level	Low income	10	3.9
	Middle income	150	58.6
	High income	96	37.5
Number of children	1 child	155	60.5
	> 1 child	101	39.5
Child’s age	< 12 months	112	43.8
	≥ 12 months	144	56.3
Child’s sex	Male	128	50.0
	Female	128	50.0
Perceived difficulty of child feeding and caregiving by mothers	Very difficult	60	23.4
	Somewhat difficult	157	61.3
	Not difficult	39	15.2
Paternal involvement in childcare	Never/Rarely	29	11.3
	Sometimes	127	49.6
	Most of the time /always	100	39.1
Presence of a caregiver for child feeding and care	No caregiver	123	48.0
	Private caregiver	49	19.1
	Grandmother	84	32.8

The findings indicated that 19.5% of mothers possessed a television in their kitchen, with 41.4% engaging in television viewing for over one hour per day on average. Among the participants, 97.3% owned a smartphone with internet access, while 40.2% had a computer and 12.1% had a tablet. The proportions of mothers using a smartphone, computer, and tablet for more than one hour per day were 87.1%, 21.8%, and 4.3%, respectively.

### Children’s exposure to digital technological devices

To facilitate childcare, 65.2% of mothers reported using at least one digital device. The most preferred device (49.6%) was the smartphone, while television (14.1%) and computer/tablet (1.6%) were used less frequently. The most common reasons for maternal use of digital devices were to soothe the baby when crying (35.9%), assist with feeding (21.1%), help the baby fall asleep (15.6%), facilitate the mother’s personal tasks (13.7%), provide educational content (11.3%), and entertain the baby with digital games (11.3%) (Table 2).

**Table 2:** Maternal use of digital devices during childcare

Variable		*n*	%
Use of any digital device	Yes	167	65.2
	No	89	34.8
Type of device used*	Smartphone	127	49.6
	Television	36	14.1
	Computer/Tablet	4	1.6
Reasons for device use**	To soothe the baby when crying	92	35.9
	To assist with feeding	54	21.1
	To help the baby fall asleep	40	15.6
	To facilitate the mother’s personal tasks	35	13.7
	To provide educational content	29	11.3
	To entertain the baby with digital games	29	11.3

^*^ Percentages are based on total sample (*n* = 256)

^**^ Mothers were allowed to select more than one response

The results showed that 60.5% of the children were exposed to television on a daily basis, with 44.1% watching for less than one hour and 16.4% for one hour or more. Additionally, 34.4% of the children were reported to rarely or occasionally watch television alone (in the absence of any other individuals, including parents or caregivers). Of the children, 53.1% used smartphone every day (41.4% < 1 h, 11.7% ≥ 1 h), 21.9% (17.6% < 1 h, 4.3% ≥ 1 h) used computer/tablet (Table 3).

**Table 3:** Children’s exposure to digital technological devices

		*n*	%
Daily television viewing time	Never	101	39.5
	< 1 h	113	44.1
	≥ 1 h	42	16.4
Watching television alone	Never	156	60.9
	Rarely/sometimes	88	34.4
	Most of the time/always	12	4.7
Daily smartphone use/exposure time	Never	120	46.9
	< 1 h	106	41.4
	≥ 1 h	30	11.7
Daily computer/tablet screen use/exposure time	Never	200	78.1
	< 1 h	45	17.6
	≥ 1 h	11	4.3

While 34.4% of mothers reported that they rarely/sometimes fed their childs in front of the television, 6.3% stated that they did so most of the time/always (total = 40.7%). These rates were 17.2% and 4.7% for computer/tablet (total = 21.9%), 37.1% and 9% for smartphone **(**total = 46.1%**)**, respectively. Additionally, 62.1% of mothers (55.9% rarely/sometimes, 6.3% most of the time/always) used a smartphone to soothe their child when crying. Furthermore, 73.8% of mothers (54.7% rarely/sometimes, 19.1% most of the time/always) played calming sounds from a smartphone to facilitate their child’s sleep.

### Factors associated with children’s exposure/use of digital technology devices

Television viewing was significantly more common among children with low paternal involvement in caregiving (66%) compared to those with high paternal involvement (52%). (*p* = 0.025). Similarly, children of mothers who watched television for one hour or more daily had a higher viewing rate (68.9%) than those of mothers who watched less (54.7%) (*p* = 0.022). Additionally, children 12 months and older had a significantly higher rate of screen viewing (73.6%) compared to younger children (43.8%), (Table 3). Logistic regression analysis showed that child age ≥ 12 months (OR = 3.69; 95% CI: 2.156–6.345), maternal TV viewing ≥ 1 h/day (OR = 1.93;95% CI: 1.110–3.365), and low paternal involvement OR = 0.554; 95% CI: 0.321–0.956) were significantly associated with higher child TV exposure (*p* < 0.05) (Tables 4 and 5).

**Table 4:** Comparison of demographic characteristics by child’s television exposure status

Characteristics		Does not watch TV (*n* = 101) *n* (%)	Watches TV (*n* = 155) *n* (%)	*p*
Mother’s age	< 30 years	40 (36.0)	71 (64.0)	0.328
	≥ 30 years	61 (42.1)	84 (57.9)	
Mother’s education	Below high school	24 (32.4)	50 (67.6)	0.143
	High school or higher	77 (42.3)	105 (57.7)	
Mother’s employment	Employed	47 (37.9)	77 (62.1)	0.623
	Unemployed	54 (40.9)	78 (59.1)	
Family income level	Low-Moderate	63 (39.4)	97 (60.6)	0.974
	High	38 (39.6)	58 (60.4)	
Number of children	1 child	66 (42.6)	89 (57.4)	0.205
	> 1 child	35 (34.7)	66 (65.3)	
Paternal involvement in childcare	Low (never/rarely/sometimes)	53 (34.0)	103 (66.0)	**0.025**
	High (most of the time/always)	48 (48.0)	52 (52.0)	
Additional caregivers	No additional caregiver	48 (39.0)	75 (61.0)	0.846
	Private caregiver	18 (36.7)	31 (63.3)	
	Grandmother	35 (41.7)	49 (58.3)	
Mother’s daily TV time	< 1 h	68 (45.3)	82 (54.7)	**0.022**
	≥ 1 h	33 (31.1)	73 (68.9)	
Perceived difficulty in child care	Very difficult/ difficult	85 (39.2)	132 (60.8)	0.827
	Not difficult	16 (41.0)	23 (59.0)	
Child’s age	< 12 months	63 (56.3)	49 (43.8)	** < 0.001**
	≥ 12 months	38 (26.4)	106 (73.6)

**Table 5:** Comparison of characteristics by child’s smartphone exposure/use status

Characteristics		No smartphone exposure/Use (*n* = 120) *n* (%)	Smartphone exposure/Use (*n* = 136) *n* (%)	*p*
Mother’s age	< 30 years	48 (43.2)	63 (56.8)	0.303
	≥ 30 years	72 (49.7)	73 (50.3)	
Mother’s education	Below high school	31 (41.9)	43 (58.1)	0.308
	High school or higher	89 (48.9)	93 (51.1)	
Mother’s employment	Employed	53 (42.7)	71 (57.3)	0.199
	Unemployed	67 (50.8)	65 (49.2)	
Family income	Low-Moderate	71 (44.4)	89 (55.6)	0.301
	High	49 (51.0)	47 (49.0)	
Number of children	1 child	74 (47.7)	81 (52.3)	0.731
	> 1 child	46 (45.5)	55 (54.5)	
Paternal involvement in childcare	Low (never/rarely/sometimes)	63 (40.4)	93 (59.6)	**0.009**
	High (most of the time/always)	57 (57.0)	43 (43.0)	
Additional caregivers	No additional caregiver	59 (48.0)	64 (52.0)	0.931
	Private caregiver	22 (44.9)	27 (55.1)	
	Grandmother	39 (46.4)	45 (53.6)	
Mother’s daily smartphone use time	< 1 h	19 (57.6)	14 (42.4)	0.187
	≥ 1 h	101 (45.3)	122 (54.7)	
Perceived difficulty in child care	Very difficult/ difficult	100 (46.1)	117 (53.9)	0.549
	Not difficult	20 (51.3)	19 (48.7)	
Child’s age	< 12 months	75 (67.0)	37 (33.0)	** < 0.001**
	≥ 12 months	45 (31.3)	99 (68.8)	

Smartphone exposure/use was significantly more common among children aged 12 months and older (68.8%) and those with low paternal involvement in caregiving (59.6%) (*p* < 0.001 and *p* = 0.009, respectively) (Table 6). Regression analysis confirmed that being aged ≥ 12 months (OR = 4.537; 95% CI: 2.651–7.765; *p* < 0.001) increased the odds, whereas high paternal involvement (OR = 0.493; 95% CI: 0.285–0.850; *p* = 0.011) reduced the odds of smartphone exposure/use.

**Table 6:** Comparison of demographic characteristics by child’s computer/tablet exposure/use status

Characteristics		No computer/Tablet exposure/Use (*n* = 200) *n* (%)	Computer/Tablet exposure/Use (*n* = 56) *n* (%)	*p*
Mother’s age	< 30 years	88 (79.3)	23 (20.7)	0.696
	≥ 30 years	112 (77.2)	33 (22.8)	
Mother’s education	Below high school	59 (79.7)	15 (20.3)	0.692
	High school or higher	141 (77.5)	41 (22.5)	
Mother’s employment	Employed	92 (74.2)	32 (25.8)	0.140
	Unemployed	108 (81.8)	24 (18.2)	
Family income	Low-Moderate	126 (78.8)	34 (21.3)	0.755
	High	74 (77.1)	22 (22.9)	
Number of children	1 child	121 (78.1)	34 (21.9)	0.977
	> 1 child	79 (78.2)	22 (21.8)	
Paternal involvement in childcare	Low (Never/rarely/sometimes)	114 (73.1)	42 (26.9)	**0.015**
	High (most of the time/always)	86 (86.0)	14 (14.0)	
Additional caregivers	No additional caregiver	99 (80.5)	24 (19.5)	0.428
	Private caregiver	35 (71.4)	14 (28.6)	
	Grandmother	66 (78.6)	18 (21.4)	
Mother’s daily computer use time	< 1 h	159 (79.5)	41 (20.5)	0.315
	≥ 1 h	41 (73.2)	15 (26.8)	
Mother’s daily tablet use time	< 1 h	195 (79.6)	50 (20.4)	**0.007**
	≥ 1 h	5 (45.5)	6 (54.5)	
Perceived difficulty in child care	Very difficult/somewhat difficult	166 (76.5)	51 (23.5)	0.137
	Not difficult	34 (87.2)	5 (12.8)	
Child’s age	< 12 months	96 (85.7)	16 (14.3)	**0.010**
	≥ 12 months	104 (%72.2)	40 (27.8)	

Computer/tablet exposure/use was significantly more common among children with low paternal involvement (26.9%), maternal tablet use of ≥ 1 h/day (54.5%), and those aged ≥ 12 months (27.8%) (*p* < 0.05 for all) (Table 5). Regression analysis confirmed that being aged ≥ 12 months (OR = 2.276; 95% CI: 1.181–4.388), maternal tablet use ≥ 1 h/day (OR = 4.095; 95% CI: 1.151–14.570), and low paternal involvement (OR = 0.472; 95% CI: 0.239–0.933) were significant predictors of computer/tablet exposure/use.

A total of 55.1% of mothers reported that they had not received any information from a physician regarding the harms of children’s exposure to digital devices.

## Discussion

This study examined maternal behaviors regarding the use of digital technological devices during child care, as well as screen exposure among young children and the factors associated with this exposure. Our findings revealed that more than half of the mothers frequently used digital devices -particularly smartphones (65.2%)- to facilitate child feeding and caregiving. This indicates the widespread integration of digital technology, especially smartphones due to their accessibility and multifunctionality, into everyday parenting practices. However, while smartphones may offer short-term convenience, they also have the potential to hinder mothers’ full engagement with their children [18]. The use of smartphones during caregiving may disrupt mother–child interactions, which could in turn diminish maternal sensitivity and negatively affect the child’s responsiveness to the mother [19]. On the other hand, when used intentionally and within appropriate limits, smartphones can serve as practical tools that help parents access useful information about childcare and health, potentially contributing to more informed and supportive parenting practices.

### Children’s screen exposure

Findings from the current study indicate that more than half of the children were exposed daily to television and smartphones, while approximately one-fifth were exposed to computers or tablets. Consistent with these results, Kulakci-Altintas reported that among 0–3-year-old children in Turkey, smartphones (40.8%) were the most frequently used devices, followed by television (39.6%) and tablets/computers (19.6%) [6].

Similarly, previous studies have reported high rates of screen exposure among young children across different countries. Slobodin et al. [20] found that more than half of Israeli infants aged 4–10 months were using screen media by 12 months of age, and over one-third had already been exposed to screen media by six months. In Spain, Paz-Kantos et al. [21] reported that 44.7% of children under the age of five used smartphones and/or tablets. In the United States, Went et al. [22] determined that 79% of children younger than 18 months had screen exposure.

These findings indicate that early screen exposure is a common phenomenon worldwide, with infants and young children frequently using digital devices. Developing age-appropriate, high-quality digital content and ensuring that screen use occurs under caregiver supervision may help minimize potential harms while promoting limited developmental benefits for young children.

### Feeding in front of the screen

Our study found that 40.7% of mothers fed their children while exposing them to television, 46.1% to smartphones, and 21.9% to computers or tablets. Similarly, a study conducted in Turkey demonstrated that approximately one-third of children under the age of two were exposed to television or tablets during mealtime [23]. A study conducted in Lithuania reported that more than half of children aged 2–5 years were exposed to screens during mealtimes. Among these children, 33.7% were exposed occasionally, such as a few times per week or month, while 22% experienced screen exposure daily or at every meal. [24]. Additionally, Tombeau Cost et al. [25] found that approximately three-quarters of children aged 7–18 months were exposed to screens daily, with one-third being exposed specifically during feeding. While screens may offer short-term convenience during mealtimes, excessive exposure may have adverse effects on child development, including altered brain connectivity [26]. Previous studies have also linked screen use during feeding to increased obesity risk in toddlers and weakened mother-infant interaction [18, 27].

### Other reasons for using digital devices in childcare

Our study identified the most common reasons for digital device use outside of feeding as soothing a crying baby, helping the baby fall asleep, and allowing the mother to attend to personal tasks. Similarly, a study conducted by Levine et al. [28] in 2018 found that parents used mobile phones for various purposes in young children, such as education, entertainment, distraction, providing relaxation time for the child or parent, and keeping the child occupied while managing other tasks. Mothers with preschool children in the UK reported that screen time was an effective way for their children to rest, relax and have quiet time [16]. Kılıç et al. [29] reported that more than half of parents with 1–60-month-old children allow their children to use mobile devices while doing their daily chores or housework. These findings suggest that digital technologies are among the strategies to cope with challenges in different areas of childcare.

### Factors associated with children’s screen exposure

Consistent with previous studies, this study found that the likelihood of children using digital technological devices increased significantly with increasing age [15, 30]. Our study also found that childs of mothers who watched television for one hour or more per day were more likely to be exposed to television. Similarly, children of mothers who used tablets for one hour or more daily had a higher likelihood of tablet use. This finding aligns with previous studies demonstrating a relationship between parental media use patterns and children’s media consumption behaviors [31, 32].

A noteworthy finding in our study was that the rates of television, smartphone, and computer/tablet use were significantly higher among children with low paternal involvement compared to those with high paternal involvement. The literature has documented the positive effects of father help and support on child health. Fathers’ involvement in childcare and active interaction with the child has been associated with better cognitive and psychological child health outcomes [33]. It has been reported that infants whose fathers participated in their care had fewer crying spells after birth, breastfeeding periods were longer and sleep patterns were more regular [34]. These findings emphasize the importance of fathers’ role in childcare and suggest that shared responsibility of parents may have positive effects on children’s behavior.

### Information about screen exposure by the physician

In our study, 55.1% of mothers reported not receiving information from their physicians about the potential harms of children’s exposure to digital devices. Similarly, Wentz et al. [22] found that 61% of mothers could not recall receiving any recommendation from pediatricians regarding their infant’s screen exposure. Vincent and Blot (2021), in a study conducted in France, found that only 22.7% of parents had discussed their child’s screen exposure with a health professional, and more than half of these discussions were initiated by the parents themselves [35]. The findings highlight the importance of physicians and health professionals advising parents about both the potentially harmful and beneficial aspects of screen exposure in young children.

## Limitations

This study has several limitations. The sample includes a relatively high proportion of well-educated, high-income mothers, all of whom live with their spouses. Although data were collected from three different family health centers to enhance diversity, the sample was still predominantly composed of highly educated participants, which limits the generalizability of the findings. The survey data were obtained through face-to-face interviews with mothers. Mothers may be vulnerable to unwanted bias in some questions in the questionnaire. Additionally, the reported screen time durations were based on maternal estimates and may not reflect actual exposure. The absence of data on fathers’ media use is another limitation, as paternal behavior may also influence children’s screen exposure. Television and smartphone use were analyzed separately, whereas tablets and computers were evaluated together due to their similar usage characteristics and the relatively lower expected use among young children. However, the purposes for which these devices are used (e.g., calming, feeding, or entertaining) may overlap. Finally, the study did not include child developmental or behavioral outcomes, and its cross-sectional design precludes causal inference.

## Conclusion

The study found that more than half of the mothers used digital devices—primarily smartphones—during childcare. Additionally, over half of the children were exposed daily to television and smartphones, while approximately one-fifth were exposed to computers or tablets. The findings suggest that active paternal involvement in childcare may serve as a protective factor against early exposure to digital technological devices. These results highlight the importance of integrating guidance on the physical and psychosocial aspects of healthy screen use into parental counseling programs within family health centers and other pediatric care settings.

## Data Availability

All data generated or analyzed during this study are not publicly available. The dataset supporting the conclusions is available upon request to the corresponding author.
